# The Government Chemist Analyzes Two of the Leading Baking Powders

**Published:** 1882-04

**Authors:** 


### THE GOVERNMENT CHEMIST ANALYZES TWO OF THE LEADING BAKING POWDERS, AND WHAT HE FINDS THEM MADE OF.

I have examined samples of "Cleveland's Superior Baking Powder" and "Royal Baking Powder," purchased by myself in this city, and I find they contain:

"Cleveland's Superior Baking Powder."

Cream of Tartar
Bicarbonate of Soda
Flour

Available carbonic acid gas 12.61 per cent., equivalent to 118.2 cubic inches of gas per oz. of Powder.

"Royal Baking Powder."

Cream of Tartar
Bicarbonate of Soda
Carbonate of Ammonia
Tartaric Acid
Starch

Available carbonic acid gas 12.40 per cent., equivalent to 116.2 cubic inches of gas per oz. of Powder.

Ammonia gas 0.43 per cent., equivalent to 10.4 cubic inches per oz. of Powder.

Note.—The Tartaric Acid was doubtless introduced as free acid, but subsequently combined with ammonia, and exists in the Powder as a Tartrate of Ammonia.

E. G. Love, Ph. D.

New York, Jan'y 17th, 1881.

The above shows conclusively that "Cleveland's Superior" is a strictly pure Cream of Tartar Baking Powder. It has also been analyzed by Professor Johnson of Yale College; Dr. Genth of the University of Pennsylvania; President Morton of the Stevens Institute; Wm. M. Habirshaw, F.C.S., Analyst for the Chemical Trade of New York, and other eminent chemists, all of whom pronounce it absolutely pure and healthful.

JAMES VICK says that the "white worm," or any other worm, in pots, may be destroyed by sticking three or four common matches down into the soil, also one or two up into the drain opening. The phosphorus on the match is certain death to animal life, and a powerful fertilizer for plants.

